# Synergistic Stress–Corrosion Cracking of S135 Drill Pipes Induced by Sulfide–Chloride Drilling Fluid

**DOI:** 10.3390/ma19081621

**Published:** 2026-04-17

**Authors:** Jinzhou Zhang, Zhunli Tan, Lihong Han, Ping Luo, Min Zhang

**Affiliations:** 1 Material Science and Engineering Research Center, School of Mechanical, Electronic and Control Engineering, Beijing Jiaotong University, Beijing 100044, China; 22110430@bjtu.edu.cn (J.Z.); zhangm@bjtu.edu.cn (M.Z.); 2 State Petroleum Drilling and Production Equipment Key Laboratory, CNPC Tubular Goods Research Institute, Xi’an 710077, China; hanlihong@cnpc.com.cn; 3 National College for Excellent Engineers, Beijing Jiaotong University, Beijing 100044, China; 25119033@bjtu.edu.cn

**Keywords:** drill pipe, crack, drilling fluid, corrosion

## Abstract

**Highlights:**

- The failure happened by co-operation of torque and local corrosion from the drill fluid.
- Mud deposition during drill pipe service exacerbates intergranular corrosion of sulfides.
- Drilling fluid infiltrated the defects and its local corrosion accelerated crack propagation.

**Abstract:**

As a key component in oil drilling, drill pipes are prone to failure in harsh operating service environments. Multiple severe cracks were identified in the S135 drill pipes following field service, with partial crack extensions of ~1 mm detected at the thread roots penetrating into the pipe wall, posing critical threats to structural integrity. This study investigated the failure mechanisms of the drill pipes and examined the potential effects of dynamic rotation on corrosion-assisted cracking. The results showed that this failure was close to the combined results of corrosion and torque. Cl^−^ and S^2−^ in the drilling fluid were the main sources of corrosive substances. Cl^−^ preferentially accumulated on the drill pipe surface, initiating localized pitting corrosion. Under applied stress, these surface pits exacerbated local stress concentration. The synergistic action of S^2−^ then promoted the transition from pitting to stress corrosion cracking. Regarding the corrosion stage, the rotational state of the drill pipe will affect the drilling fluid’s corrosion results. The mud deposition during rotation leads to severe intergranular corrosion, which further causes material peeling. Dynamic rotation at 60 r·min^−1^ increased the corrosion rate to 0.55 mm·a^−1^ after 216 h of immersion, 41% higher than under static conditions, while maximum corrosion depth increased from 8.43 μm to 13.86 μm. These results indicate that rotational motion accelerates corrosion-assisted cracking.

## 1. Introduction

Drill pipes are the main component of the drill string, consisting of a joint and body (i.e., a pin and box) [1]. Their key function is to transmit the drilling torque via connecting tools and ground equipment. In the context of drill stem applications, the most common API steel grades include G105, S135, and analogous specifications, where the numerical designation indicates the minimum yield strength in ksi. These steels are classified as Cr-Mo steels [2]. They are generally exposed to complex stress states during services, such as alternating stress, bending, and vibration loads [3,4].

**Figure 1:**
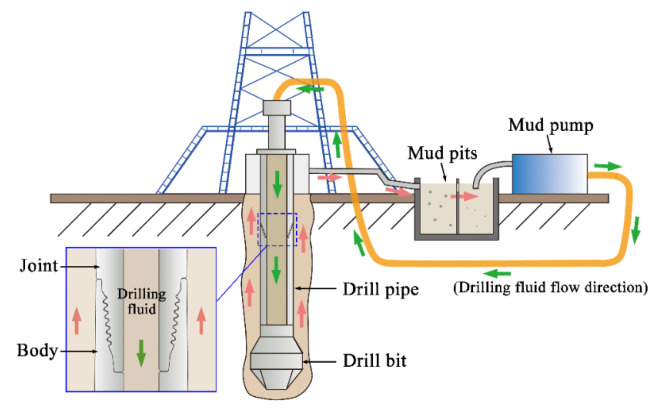
Schematic figure of drilling fluid circulation system.

The service environment of drill pipes presents an equally severe corrosive attack [5]. During drilling operations, formation fluids containing acidic gases (CO_2_ and H_2_S) readily dissolve into the circulating drilling fluid, particularly under high-temperature high-pressure (HTHP) conditions. Furthermore, the drilling fluid circulation system required during drill pipe service (Figure 1) introduces dissolved oxygen into the drilling fluid, which synergistically interacts with chloride ions to accelerate corrosion [6]. When superimposed on these corrosive conditions, mechanical loading may initiate stress corrosion cracking (SCC) and precipitate catastrophic brittle failures with minimal macroscopic deformation, posing significant safety risks [7].

Previous investigations have extensively characterized drill pipe corrosion under static immersion conditions. Zheng et al. [8] evaluated the corrosion behavior of S135, G105, and V150 steels in simulated wellbore environments, demonstrating that all three grades exhibited severe CO_2_ corrosion, while S135 and V150 additionally suffered a localized H_2_S attack. Notably, S135 exhibited the highest susceptibility to SCC at the wellhead, attributable to the synergistic interaction of pitting and tensile stress. Furthermore, the coupling of corrosion and fatigue has emerged as a critical failure mechanism. Han et al. [9] reported that S135 steel exhibited substantially higher corrosion-fatigue sensitivity (74.7%) compared with G105 (64.6%) under H_2_S-containing conditions. Similarly, Yu et al. [10] observed that the fatigue life of the V150 drill pipe in H_2_S-containing mud was reduced to merely 10% of that in air, accompanied by a ductile-to-brittle fracture transition.

While these studies provide valuable insights into static corrosion mechanisms, existing investigations have predominantly focused on static immersion scenarios, leaving the role of dynamic rotation on corrosion-assisted cracking poorly understood. Although the deleterious effects of H_2_S and Cl^−^ on drill pipe integrity under such static conditions have been widely reported, the influence of drill pipe rotation—an inherent characteristic of rotary drilling—on corrosion-assisted cracking remains inadequately characterized. The substantial alterations in corrosion behavior and crack evolution induced by drilling fluid flow and alternating stresses associated with pipe rotation warrant particular consideration.

Motivated by a recent catastrophic in-service cracking incident involving S135 drill pipes, this study conducts a comprehensive failure analysis integrating macroscopic fracture examination and cracking characterization to elucidate the causes of the accident. Subsequently, the influence of dynamic rotation on corrosion behavior is evaluated through a simulated drilling process under controlled laboratory conditions. Additionally, reasonable suggestions for preventing similar drill pipe failures were proposed.

## 2. Background, Methods, and Materials

**Figure 2:**
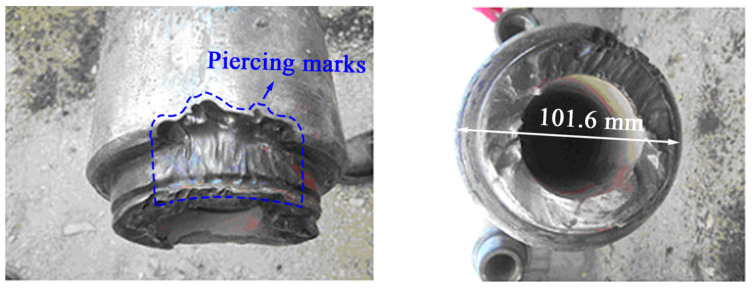
The morphologies of the drill pipe fracture.

S135 high-strength drill pipes (Φ101.6 × 9.65 mm) were involved in a joint fracture accident during the drilling process, and the fracture surface is presented in Figure 2. The fracture is located on the second thread of the joint where piercing marks existed in the surface, and there is no obvious plastic deformation. The chemical composition, impurity degree, and mechanical properties of the drill pipe were examined, with the results listed in Table 1 and Table 2, respectively. Both of them complied with the related standard requirements of API Spec 5DP-2020 [11].

This oil well was designed for a depth of 6950 m and the drill pipe fracture happened at 6780 m. Drilling parameters before the failure are as follows: weight on bit was 20–30 kN, rotation speed was 10 r·min^−1^, pump pressure was 12 MPa, and mud density was 1.98 g/cm^3^. Surface cracks were in the bottom of threads on most joints by magnetic powder flaw inspection. Drill pipes exhibited surface cracks at multiple locations such as the thread bottom and sealing surface and failures were analyzed here.

**Figure 3:**
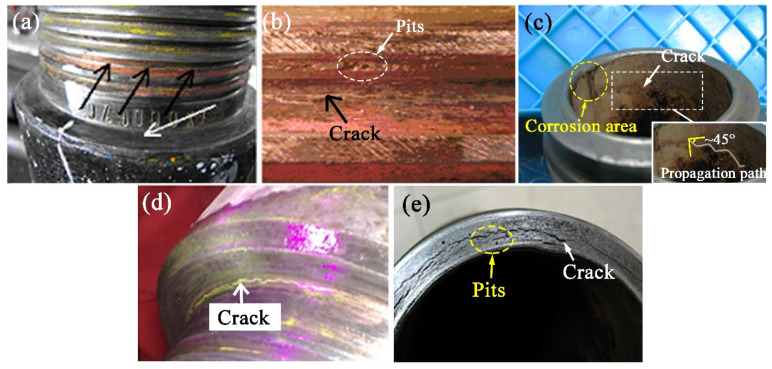
The morphologies of the drill pipe joint: (**a**) crack location indicated by the arrows, (**b**) cracks at the thread bottom, and (**c**) the corresponding inner wall; (**d**) magnetic powder flaw inspection in the main shoulder; (**e**) secondary shoulder surface.

Cracks and pits were observed in the shoulder and thread bottom regions of the drill pipe, as indicated by the white and black arrows in Figure 3a, respectively. The crack extends along the thread bottom (Figure 3b). Corresponding to the cracking thread, bottom cracks in the inner wall were found to propagate along about a 45° angle over the radial direction (Figure 3c). This is the typical crack propagation path under the operating torque of the drill pipe. Cracks propagate from the thread bottom and eventually penetrate the whole drill pipe joint. Corrosion traces in large areas were observed on the inner wall (Figure 3c). Figure 3d presents cracks at the main shoulder root detected by magnetic powder flaw inspection. On the secondary shoulder shown in Figure 3e, cracks propagate in the circumferential direction, and some pits can be seen. This indicates that the sealing surface is generating extremely high radial compressive stresses, resulting in severe cracking due to metal rheological characteristics.

S135 drill pipes were analyzed by an optical microscope (OM, Scpoe.A1, Carl Zeiss Microscopy GmbH, Jena, Germany), physical and chemical inspection, scanning electron microscopy (SEM, Zeiss EVO18, Carl Zeiss Microscopy GmbH, Jena, Germany), and energy-dispersive spectroscopy (EDS, X-Max 80, Oxford Instruments, High Wycombe, UK) techniques.

**Figure 4:**
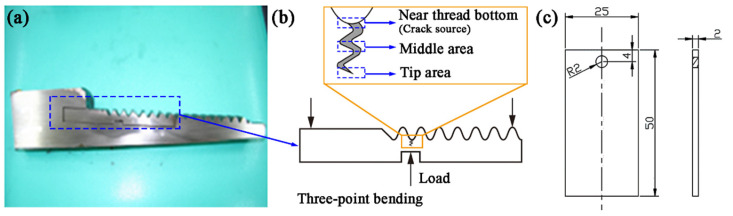
(**a**,**b**) Schematic diagram of three-point bend and crack section areas; (**c**) dimensions of immersion test samples (unit: mm).

The crack section is prepared to investigate the cracking behavior by the three-point bending method as follows: firstly, the cracked thread specimens were mechanically cut down according to the shape shown in Figure 4a; then, the specimens were loaded until fracture (Figure 4b). Three crack sections near the thread bottom, middle, and tip were observed. Those locations are also schematically illustrated in Figure 4b.

Finite element analysis (FEA) was performed using DEFORM-2D software (V10.2) to investigate the stress concentration behavior at the threaded connection of the S135 drill pipe. A two-dimensional axisymmetric model was established based on the drill pipe joint geometry (Figure 4a), comprising the joint and body. The mesh was constructed using quadrilateral elements with adaptive remeshing (maximum element size: 20 mm), employing local refinement at thread roots (minimum element size: 1 mm). The S135 steel was modeled as an elastic–plastic material with Young’s modulus E = 210 GPa and Poisson’s ratio ν = 0.30. Boundary conditions were established by fully constraining the bottom surface of the body (U_X_ = U_Y_ = 0) while applying a uniform upward axial displacement of 0.6 mm (U_Y_ = 0.6 mm) to the joint.

Immersion tests were carried out to investigate the effect of drilling fluid on the corrosion of the S135 drill pipe. Samples for immersion were cut from the S135 drill pipe, and the dimensions are displayed in Figure 4c. The operational drilling fluid is a polysulfide system mud, and its chemical composition extracted from the accident well is shown in Table 3. Considering that 60 r·min^−1^ represents a common rotational speed for drill pipes in service [12,13], a relative rotational speed of 60 r·min^−1^ between the drill pipe and drilling fluid was set to comparatively investigate the different corrosion behaviors during rotation and stationary states. These rotating and stationary samples were named 60 rpm and 0 rpm, respectively. The immersion durations for the 60 rpm and 0 rpm samples were set as 72 h, 144 h, and 216 h, with three parallel experiments conducted for each time point. After 216 h of immersion, the three-dimensional surface morphology of the samples was characterized using white light interferometry (ZYGO Nexview, ZYGO Corporation, Middlefield, CT, USA) over an area of 1668.769 μm × 1668.769 μm. The average surface roughness (*S*_a_) and maximum corrosion depth (*S*_z_) were subsequently quantitatively analyzed.

The corrosion rate *V* (mm·a^−1^) of the 0 rpm and 60 rpm samples after immersion for 72 h, 144 h, and 216 h was calculated using Equation (1) [14]:

(1) $$V=\frac{\left(w_{0}-w_{1}\right)\times 87,600}{\rho \times A\times t}$$

where *ρ* is the material density (g·cm^−3^), *A* is the specimen surface area (cm^2^), *t* is the corrosion time (h), and *w*_0_ and *w*_1_ are the specimen masses (g) before and after corrosion, respectively, as measured using an electronic balance with 0.1 mg precision.

## 3. Results and Discussion

### 3.1. Cracking Analysis

**Figure 5:**
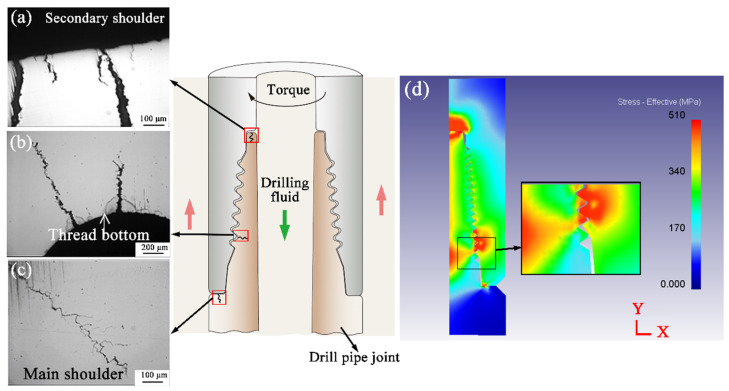
Schematic diagram of drill pipe joint cracking location and cross-sectional morphologies of the cracks at the (**a**) secondary shoulder, (**b**) thread bottom, and (**c**) main shoulder root; (**d**) stress distribution nephogram of drill pipe.

From the inspection results of the drill pipe joints in Figure 3, the main shoulder, secondary shoulder, and thread bottom are three major areas of cracking (schematically show in Figure 5). Their cross-sectional morphologies are illustrated in Figure 5a–c. Figure 5a presents the wider cracks at the secondary shoulder compared with the thread bottom and main shoulder, which agree with the crack in Figure 3e. The cracks were initialized at the thread bottom surface and penetrated ~1 mm into the metal matrix (Figure 5b). Figure 5c displays a fine crack on the main shoulder root (white arrow location in Figure 3a).

For all the failures of drill pipes investigated in this work, their deformation was found on the sealing surface of the secondary shoulder (Figure 3e). The deformation characteristics indicate that there exists excessive torque during the drilling process. Compressive stress will be caused by the axial direction action of the thread helix angle during this torque. In order to study the cross-sectional stress distribution in the connection between the drill pipe joint and body, the FEA model is adopted. Related compressive stress distribution is obtained by simulating the loading process of the drill pipe in axial movement (U_Y_ = 0.6 mm). The effective stress distribution of the drill pipe joint after simulation is displayed in Figure 5d. There are different degrees of stress concentration at the thread bottom and joint shoulder. The maximum effective stress exceeded 500 MPa at the second engaged thread root of the joint. This stress corresponds to above ~50% of the yield strength of S135 steel, indicating that the connection remains in the elastic regime. The local stress elevation reflects the geometric stress concentration at the thread bottom, with the thread root experiencing approximately two times the nominal pipe body stress. Relatively high stress concentration positions occur at the thread root and shoulder surface throughout the entire drill pipe, which is consistent with the actual cracking locations in the drill pipe shown in Figure 3. Similar results have also been reported in the Refs. [15,16].

### 3.2. Crack Section Analysis

**Figure 6:**
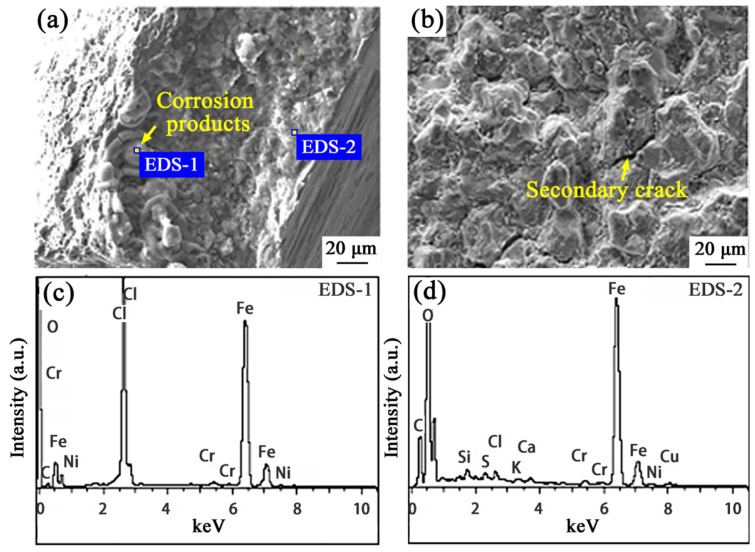
The morphologies of crack sections: (**a**) near the thread bottom surface and (**b**) middle area; EDS spectra at positions (**c**) EDS-1 and (**d**) EDS-2 indicated in (**a**).

Fracture samples were taken in situ at the cracked thread bottom of the drill pipe joint (Figure 4a) to observe the crack cross-section surface here. The crack section near the thread bottom surface is covered with corrosion products (Figure 6a). It is speculated that this is caused by the sealing face destruction, making the drilling fluid contact the thread bottom and accumulate in microcracks. For the crack middle area (Figure 6b), corrosion products are not obvious and a secondary crack with an intergranular feature also exists.

To investigate the main element characteristic of the crack section near the thread bottom surface, EDS analysis was performed on the positions indicated in Figure 6a. Specifically, EDS-1 and EDS-2 positions correspond to the corrosion product and material matrix, respectively. Meanwhile, the corresponding EDS spectra are presented in Figure 6c,d. Compared with the matrix, these corrosion products exhibit a high chloride ion concentration. Chloride ions tend to aggregate at initial microcrack locations [17,18], and the accumulated Cl^−^ greatly aggravates cracking under the co-existence of stress concentration and corrosive media.

**Figure 7:**
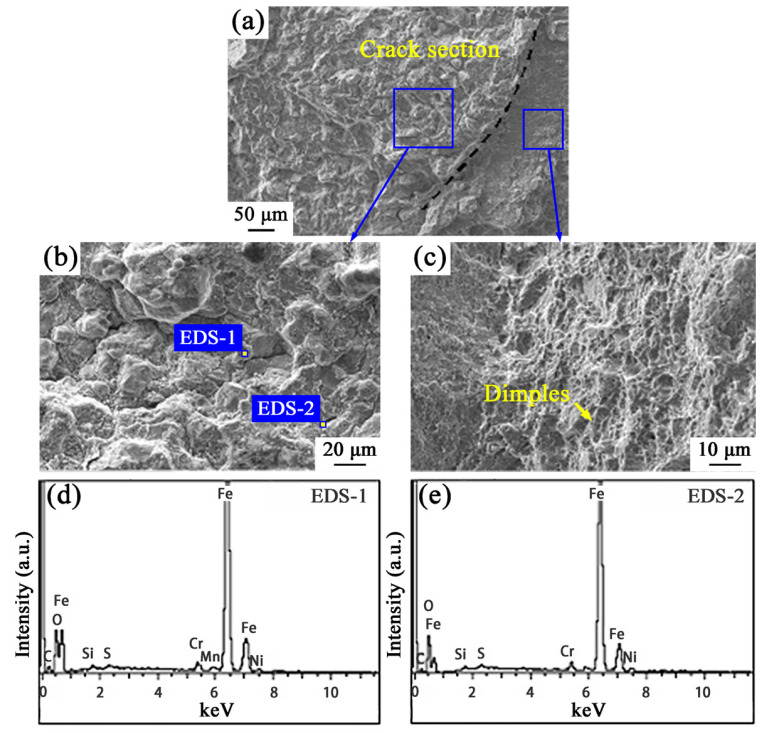
The morphologies of the fracture surface: (**a**) crack section near the tip; the magnification of (**b**) the crack tip and (**c**) fresh fracture area in (**a**); EDS spectra at positions (**d**) EDS-1 and (**e**) EDS-2 indicated in (**b**).

Morphology of the crack tip area on the failure of the drill pipe joint is displayed in Figure 7. The left side of the dashed line in Figure 7a stands for the crack section part, and the right side is the fresh fracture area by three-point bending. Their local magnified morphologies are presented in Figure 7b,c, respectively. The fresh fracture area (Figure 7c) exhibits obvious tough features with many dimples in contrast to the brittle intergranular characteristics of the crack section (Figure 7b). Related EDS results of the corresponding crack tip section (left side part of Figure 7a) are shown in Figure 7d,e and Table 4. There is obviously an amount of sulfur that existed almost without the chlorine element in the tip area, which is different from that in the crack section near the thread bottom (Figure 6c,d). Obviously, there are lots of chlorine elements without sulfur in the latter one. This indicates the possible occurrence of sulfide corrosion near the crack tip.

There are four possible main reasons for brittle intergranular fracture of high-strength drill pipe steels: (i) segregation of impurity elements (phosphorus, etc.) [19], (ii) presence of brittle inclusions (Al_2_O_3_, TiN, etc.) at grain boundaries [20,21], (iii) over-heating or over-burning defects [22], and (iv) the co-operation of corrosion and stress [23]. Based on the above analysis results about the manufacturing process, microstructure, mechanical properties, service condition, etc., the most plausible causes for the S135 drill pipe fracture here are the combined effects of corrosion and stress.

S135 drill pipes with high strength and hardness are susceptible to cracking induced by corrosion and stress below the yielding strength. The time for brittle fracture to happen varies from minutes to years, depending on the service conditions and the stress state. Here, the combination of corrosive media and stress concentration in service is a key promoting factor for S135 fracture. Based on the crack morphology analysis, the cracking of the S135 drill pipe can be interpreted through a synergistic mechanism: Cl^−^ induces pitting corrosion and microcrack nucleation, creating localized stress concentration zones [24]; subsequently, S^2−^ drives SSC by adsorbing at pit and microcrack sites and retarding hydrogen recombination, thereby promoting hydrogen absorption and embrittling the steel matrix [25]. Finally, applied stress facilitates crack propagation through the hydrogen-weakened region, leading to ultimate failure of the S135 drill pipe under high-temperature high-pressure drilling conditions.

### 3.3. Drilling Fluid Corrosion Characterization

**Figure 8:**
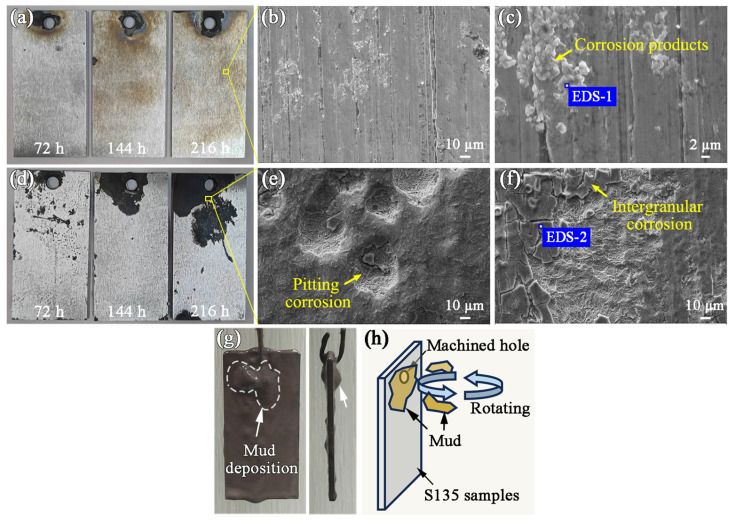
Surface morphology of drill pipe samples after the immersion test: (**a**–**c**) 0 rpm and (**d**–**f**) 60 rpm samples; (**g**) front and side views of mud deposition morphology on 60 rpm samples before cleaning; (**h**) schematic diagram of mud deposition.

Apparent corrosion characteristics were found in the crack analysis described previously. The drilling fluid’s corrosive effects on the S135 drill pipe were verified according to the immersion test set up in the above Methodology Section. The variation in corrosion between rotating and stationary drill pipe samples is considered. The macroscopic corrosion morphologies of the 0 rpm and 60 rpm samples immersed for 72 h, 144 h, and 216 h are shown in Figure 8a,d, respectively. The surface of the 0 rpm sample exhibits obvious uniform corrosion on the surface after immersion for 216 h. The surface covered with corrosion products was observed after magnification. Meanwhile, the corrosion deepened at the sample machined marks, which was due to the tendency of Cl^−^ to accumulate at the defects (Figure 8b,c). EDS analysis results of corrosion products are shown in Table 4. The corrosion of 0 rpm samples was mainly caused by Cl^−^.

For the 60 rpm samples, more distinct corrosion areas were found after 216 h of corrosion, and the corrosion areas were apparently enlarged with the increase in corrosion time. The morphologies of the corrosion region of 60 rpm samples after 216 h of immersion are shown in Figure 8e,f. Corrosion pits of ~40 μm in diameter are observed on the surface of these samples (Figure 8e), in addition to surface peeling and intergranular corrosion features (Figure 8f). EDS point analyses were performed at positions EDS-1 and EDS-2, as indicated in Figure 8c,f, and the results are listed in Table 5. From Table 5, it was found that the corroded area contained a high content of S^2−^. Furthermore, it is noteworthy that corrosion for 60 rpm (Figure 8d) tends to extend near the sample machined holes, and visible mud deposition occurs at the sample machined holes before cleaning, as demonstrated in Figure 8g. This deposition corresponds to the corrosion region, meaning that mud deposition is inferred to exacerbate sample corrosion in the rotation condition. The less fluid mud tends to accumulate at the machined holes, while the mud deposition volume is gradually enlarged with a rotary immersion time increment, as depicted schematically in Figure 8h. Accordingly, the corrosive effect of mud deposition needs to be considered.

**Figure 9:**
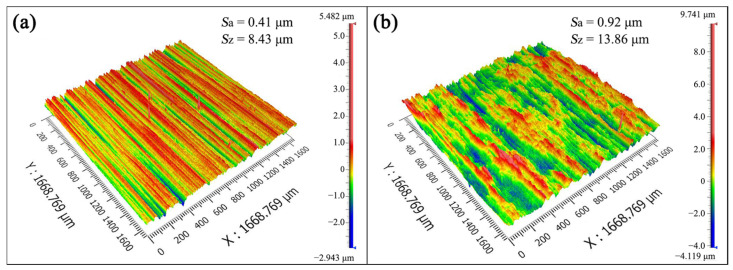
Three-dimensional surface morphologies of (**a**) 0 rpm and (**b**) 60 rpm samples after 216 h of immersion.

The three-dimensional surface morphologies of the 0 rpm and 60 rpm samples after 216 h of immersion are shown in Figure 9a,b, respectively. The average surface roughness *S*_a_ and maximum corrosion depth *S*_z_ of the 60 rpm sample were 0.92 μm and 13.86 μm, respectively (Figure 9b), both showing increases compared to the 0 rpm sample (Figure 9a). This indicates that mud deposition induced by dynamic rotation accelerates surface corrosion.

**Figure 10:**
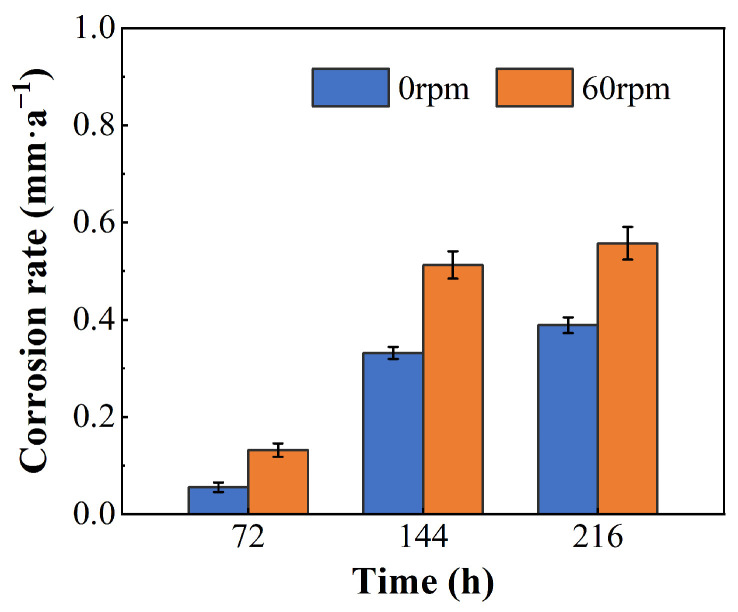
Corrosion rates of 0 rpm and 60 rpm samples after different immersion times.

The corrosion rates of the 0 rpm and 60 rpm samples after different immersion times are shown in Figure 10. Both samples exhibited relatively low corrosion rates after 72 h of immersion, at 0.06 mm·a^−1^ and 0.13 mm·a^−1^, respectively. As the immersion time extended, the corrosion rates gradually stabilized. After 216 h of immersion, the corrosion rate of the 60 rpm sample reached 0.55 mm·a^−1^, which was 41% higher than that of the 0 rpm sample. Compared with the 0 rpm sample, the 60 rpm sample exhibited a faster corrosion rate.

**Figure 11:**
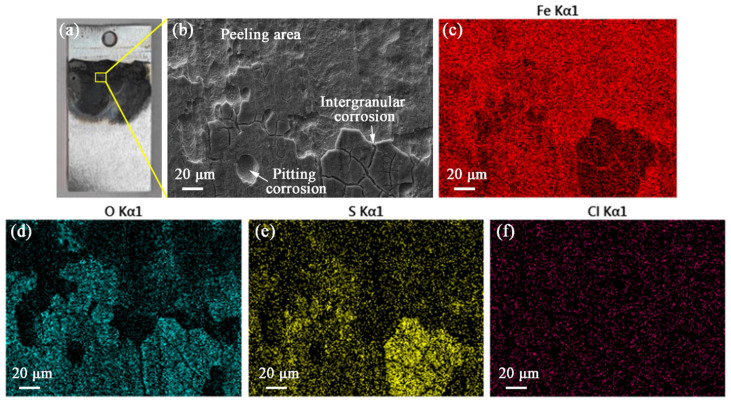
(**a**,**b**) Mud immersion corrosion morphology; EDS mapping results of (**c**) Fe, (**d**) O, (**e**) S, and (**f**) Cl elements.

Sample immersion tests were done in an environment consisting mostly of deposited drilling mud to verify the corrosive effects. Obvious corrosion areas after 216 h of immersion are observed (Figure 11a). These samples exhibited similar characteristics to the 60 rpm samples, including pitting and intergranular corrosion, as illustrated in Figure 11b. However, aggravated intergranular corrosion could lead to peeling of the material. EDS mapping results of the corrosion surface are shown in Figure 11c–f. It is noteworthy that obvious S element aggregation occurs in the intergranular corrosion region. This indicates that sulfide corrosion is the main medium causing intergranular corrosion. In addition, the O element mainly displayed in the unpeeled area could result from the incomplete destruction of the surface passivation film (Figure 11d).

The composition of the drilling fluid from Table 3 exhibits a high content of the S element. Its degradation is a possible source of sulfur ions due to polysulfide compounds in drilling fluid. Drilling fluids contain polysulfides, and their degradation is the main source of S^2−^. Moreover, the pH value of drilling fluid will decrease to weak acidity under an HTHP environment. This thermal degradation might result in the release of H_2_S, CO_2,_ and/or CO to contaminate the drilling fluid. A weak acid solution environment is formed when H_2_S is dissolved in fluid, which corrodes the alloy matrix in turn and produces ferric sulfide (FeS_x_). The reaction equation is as follows [26]:

(2) $$H_{2}S+Fe\to {FeS}_{x}+2H^{+}$$

Ferrous disulfide is a typical corrosion product in an acidic environment containing sulfur ions, especially under environmental conditions such as high temperature and low concentration of hydrogen sulfide. In addition, hydrogen sulfide dissociates to produce hydrogen ions and sulfides as described by the following Equations (3) and (4) [27]:

(3) $$H_{2}S\to H^{+}+{HS}^{-}$$

(4) $${HS}^{-}+{OH}^{-}\to S^{2-}+H_{2}O$$

Sulfide corrosion failure behavior is mainly caused by H^+^ and HS^−^ in Equation (3). The hydrogen produced by the reaction of H_2_S with the drill pipe material diffuses into the crystal lattice and dislocation as well as forms a hydrogen-enriched region. This leads to increased crack propagation rates and brittle fracture [28]. The grain boundaries are usually accompanied by a high density of dislocations. Thus, sulfides tend to induce intergranular corrosion, which further leads to material fracture. This is consistent with the brittle fracture characteristics of the drill pipe caused by sulfides observed in Figure 7b,d.

### 3.4. Failure Drill Pipe Cracking Mechanism

**Figure 12:**
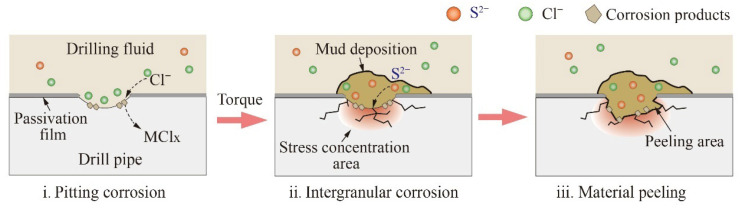
Cracking failure mechanism of the drill pipe.

Based on the above analysis, this drill pipe cracking is divided into three stages, and the schematic mechanism is shown in Figure 12.

Stage 1: Cl^−^ in drilling fluid combines with metallic elements such as Cr and Fe to form chloride (MCl_x_) [29]. This causes a destructive effect on the passivation film, which commonly exists at the boundaries of the matrix and solution. Then, an electric couple anode can be formed in those destroyed passivation film areas, and the undestroyed areas become the electric couple cathode. This is a passivation–activation cell system exacerbating material pitting corrosion. Hence, corrosion products near the pits mainly contain Cl^−^ (Figure 6a).

Stage 2: Drill pipe rotation will deposit mud during its service (Figure 8d,g). Uneven material surfaces exacerbate deposition, such as corrosion pits and surface notches of a drill pipe. The deposited mud is a locally enclosed area that enables S^2−^ to further conduct intergranular corrosion. In addition, the drilling fluid circulation is an open system (Figure 1). The devices such as mud pit and pumps inevitably dissolve O_2_ into the drilling fluid during operation. However, the dissolved O_2_ in that mud is in full contact with the drill pipe surface, thereby exacerbating oxygen corrosion. Under torsional loading, stress concentrations develop at pre-existing microcracks or defects within the drill pipe, thereby facilitating crack initiation.

Stage 3: The material is prone to peel along the grain boundaries and material integrity is destroyed with the aggravation of intergranular corrosion. At the same time, this also leads to a decrease in material strength, exacerbating the crack propagation process. Finally, brittle cracking occurs in the S135 drill pipe under the action of S^2−^.

## 4. Conclusions

The present work analyzes the causes of failure in S135 drill pipes. The main conclusions are as follows:

(1) The failure of the S135 high-strength drill pipe was related to the co-operation of corrosion and torque. The high stress concentration areas were induced by formed pitting corrosion, which create potential sites for crack initiation.
(2) Drilling fluid mud tends to be deposited at locations such as corrosion pits and surface notches during drill pipe rotation. Sulfides within the mud exacerbate intergranular corrosion. Attention needs to be paid to corrosion protection in the mud deposition areas.
(3) Under simulated drill pipe rotation at 60 r·min^−1^, the corrosion rate calculated after 216 h of immersion in drilling fluid reached 0.55 mm·a^−1^, representing a 41% increase compared to stationary conditions. Additionally, the maximum corrosion depth increased from 8.43 μm to 13.86 μm. Therefore, dynamic rotation significantly accelerates drill pipe corrosion. Future studies on drill pipe corrosion are recommended to incorporate rotational speed effects to better simulate actual service environments.
(4) Appropriate drilling fluid containing less Cl^−^ content should be used, especially in HTHP environments. In addition, excessive torque should be avoided to prevent rapid cracking induced by S^2−^.

## Figures and Tables

**Table 1:** Chemical composition of S135 drill pipe material (wt.%).

C	Si	Mn	P	S	Cu	Ni	Cr	Mo	Al	Fe
0.38	0.28	0.75	0.003	0.002	0.009	1.02	1.05	0.36	0.02	Balance

**Table 2:** Mechanical properties of S135 drill pipe.

Yield Strength (MPa)	Tensile Strength (MPa)	Elongation (%)	Area Reduction (%)	Impact Energy (A_KV2_, J)	Hardness (HBW)			Inclusions
					Outer	Middle	Inner	
1021 ± 5	1131 ± 4	21 ± 1	60 ± 1	88 ± 2	330 ± 4	323 ± 3	325 ± 3	D 0.5, others 0

**Table 3:** Chemical analysis result of drilling fluid (wt.%).

Elements	S	Cl	K	Na	Ca	Mg	Zn	Cu	P
Concentration	2.31	0.27	0.40	8.15	6.35	0.45	0.01	0.02	0.12

**Table 4:** EDS analysis of the crack section (wt.%).

Elements	C	O	Si	S	Cr	Fe	Ni
EDS-1	0.4	8.7	0.5	0.3	1.6	86.8	1.7
EDS-2	0.3	9.2	0.4	0.5	1.7	85.7	2.2

**Table 5:** EDS analysis of corrosion products (wt.%).

Elements	O	Cl	S	Ca	Mn	Cr	Fe
EDS-1	11.5	0.5	0.3	0.2	0.3	1.1	86.1
EDS-2	23.6	-	1.2	0.1	0.5	1.0	73.6

## Data Availability

The original contributions presented in this study are included in the article. Further inquiries can be directed to the corresponding author.

## Abbreviations

The following abbreviations are used in this manuscript:

HTHP	High temperature and high pressure
SCC	Stress corrosion cracking
OM	Optical microscope
SEM	Scanning electron microscopy
EDS	Energy-dispersive spectroscopy
FEA	Finite element analysis
*S*_a_	Average surface roughness
*S*_z_	Maximum corrosion depth
